# Supplementary figures and images for: Insights into the phylogenetic relationships and species boundaries of the Myricaria squamosa complex (Tamaricaceae) based on the complete chloroplast genome
Source: PeerJ. 2023 Dec 11;11:e16642. doi: 10.7717/peerj.16642 (PMC10720482; doi:10.7717/peerj.16642)

- The *M. squamosa* complex (P1)
- The *M. squamosa* complex (P2)
- *M. laxiflora* (P3)
- *M. wardii* (P4)
- *M. rosea* (P5)
- *M. elegans* (P6)

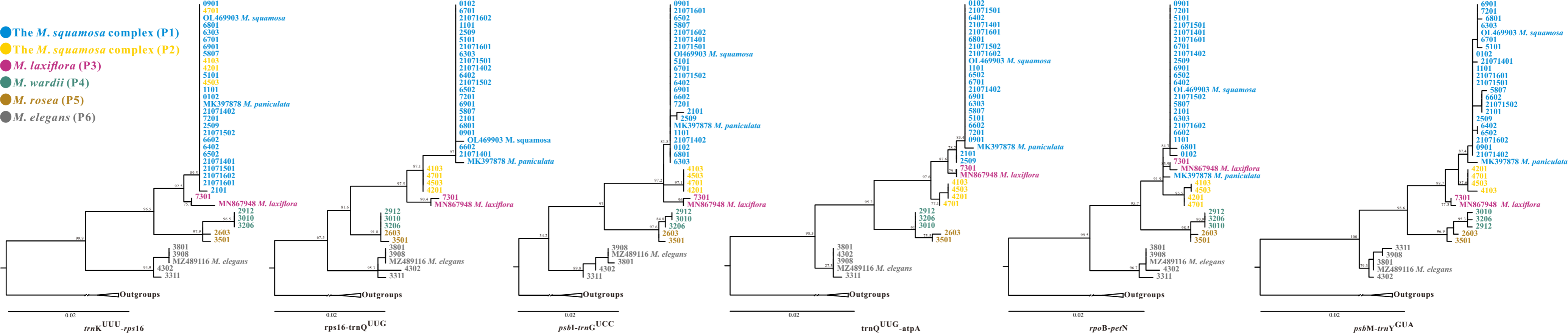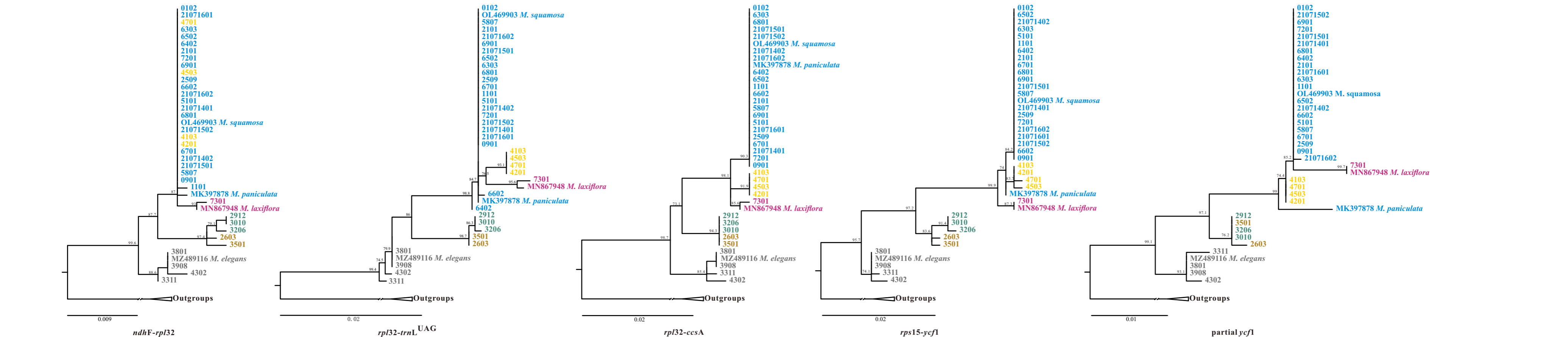

Supplement: Supplemental Information 1 — The sample codes, represented by 4–8 digits, are assigned to the terminals on the cladogram are sample codes. Different colors are used to indicate putative phylogenetic “species” (P1–P6) that were delimited through plastome phylogenetic analyses. [file peerj-11-16642-s001.pdf]
